# 461. COVID-19 Hospitalizations among Vaccinated and Unvaccinated Immunocompromised Children, 23 States, December 2022 – April 2023

**DOI:** 10.1093/ofid/ofad500.531

**Published:** 2023-11-27

**Authors:** Angela P Campbell, Laura D Zambrano, Margaret M Newhams, Michael J Wu, Regina Simeone, Amber Orzel, Natasha B Halasa, Katherine E Fleming-Dutra, Adrienne G Randolph

**Affiliations:** Centers for Disease Control and Prevention, Atlanta, Georgia; Centers for Disease Control and Prevention, Atlanta, Georgia; Boston Children's Hospital, Boston, Massachusetts; Centers for Disease Control and Prevention, Atlanta, Georgia; Centers for Disease Control and Prevention, Atlanta, Georgia; Boston Children's Hospital, Boston, Massachusetts; Vanderbilt University Medical Center, Nashville, Tennessee; Centers for Disease Control and Prevention, Atlanta, Georgia; Boston Children's Hospital, Harvard Medical School, Boston, Massachusetts

## Abstract

**Background:**

Immunocompromised (IC) persons are at increased risk of severe COVID-19 and can have a suboptimal immune response to a primary 2-dose COVID-19 vaccine series; thus, vaccination recommendations for IC persons include additional COVID-19 vaccine doses. We describe outcomes and vaccination status among US IC children hospitalized with laboratory-confirmed COVID-19 during a period of SARS-CoV-2 Omicron circulation.

**Methods:**

Children with and without an IC condition hospitalized for COVID-19 were enrolled as case-patients into a vaccine effectiveness investigation as part of the Overcoming COVID-19 network of 31 hospitals across 23 states. Data on IC conditions abstracted from the medical record included the presence of an “active or prior oncologic disorder” or a “non-oncologic immunosuppressive disorder.” Results for IC case-patients with COVID-19 enrolled from 12/18/2022–4/3/2023 (Omicron period) were analyzed by age group and vaccination status.

**Results:**

Of 239 IC case-patients, 127 (53%) had an oncologic disorder and 112 (47%) non-oncologic; 120 (50%) had > 1 additional underlying medical condition (Table 1). Thirty (13%) IC case-patients were aged 8 months–4 years, 115 (48%) 5–11 years, and 94 (39%) 12–18 years. Life-threatening illness or death occurred in 1 (3%) IC case-patient aged 8 months–4 years, 8 (7%) aged 5–11 years , and 13 (14%) aged 12-18 years (including 2 deaths). Among 209 IC case-patients aged 5–18 years, 26 (23%) of those with oncologic and 27 (28%) with non-oncologic disorders had received 2 doses of a monovalent primary COVID-19 vaccine series; 6 (5%) and 14 (14%) had received > 3 doses, respectively (p=.03; Table 2). A significantly higher proportion of IC case-patients (n=73/209; 35%) had received at least 2 COVID-19 vaccine doses compared with non-IC patients (n=284/1021; 28%; p=0.04; Table 2).

**Figure:**
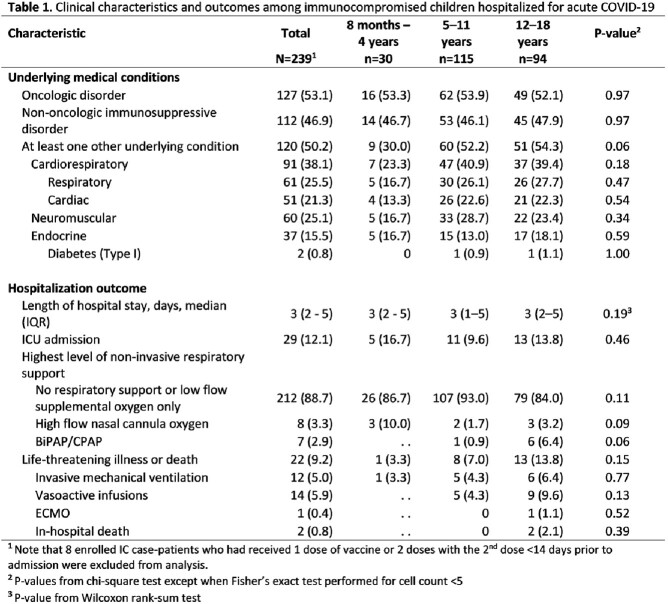

**Figure:**
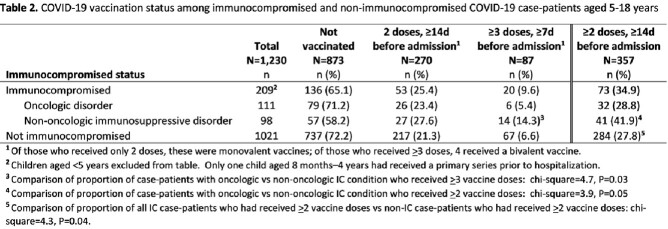

**Conclusion:**

During Omicron, immunocompromised children hospitalized for COVID-19 were more frequently aged > 5 years and > 99% survived. Vaccination coverage was low among all case-patients. A higher proportion of IC children who received >2 vaccine doses were among those case-patients hospitalized for COVID-19 compared with non-IC children. Additional doses to improve protection in IC children are recommended.

**Disclosures:**

**Regina Simeone, PhD**, Pfizer: Stocks/Bonds **Natasha B. Halasa, MD, MPH**, Merck: Grant/Research Support|Quidell: Grant/Research Support|Quidell: donation of kits|Sanofi: Grant/Research Support|Sanofi: vaccine support

